# Outcomes of primary membranous nephropathy based on serum anti-phospholipase A2 receptor antibodies and glomerular phospholipase A2 receptor antigen status: a retrospective cohort study

**DOI:** 10.1080/0886022X.2020.1792315

**Published:** 2020-07-17

**Authors:** Peihong Yin, Junxian Wang, Wenyi Liang, Linda Zhan, Yuanhao Liu, Jian Lin, Xiaohong Chen, Yingqin He, Hongyun Jian, Zhibin Xie, Xiaofan Tan, Qing Ye, Fengxian Huang

**Affiliations:** a Department of Nephrology, Zhongshan City People’s Hospital, Zhongshan, Guangdong, China; b Department of Nephrology, The First Affiliated Hospital, Sun Yat-sen University, Guangzhou, Guangdong, China

**Keywords:** Outcome, p, rimary membranous nephropathy, phospholipase A2 receptor, remission

## Abstract

**Introduction:**

Primary membranous nephropathy (PMN) is associated with the anti-phospholipase A2 receptor (anti-PLA2R) antibody in 70% of cases. Some anti-PLA2R-negative patients have the PLA2R antigen in renal tissue. This study examined the prognosis of patients with PMN according to their serum anti-PLA2R antibody (SAb) and glomerular PLA2R antigen (GAg) status.

**Methods:**

Patients diagnosed with PMN were included retrospectively. Patients were grouped according to their PLA2R status into the SAb−/GAg−, SAb−/GAg+, and SAb+/GAg + groups. Baseline data, renal biopsy results, treatment, and clinical data were compared among the groups. Cox univariable and multivariable analyses examined the factors related to complete remission (CR).

**Results:**

A total of 114 patients were enrolled; 10 (9%) in the SAb−/GAg−, 23 (20%) in the SAb−/GAg+, and 81 (71%) in the SAb+/GAg+ groups. Cumulative CR rate showed a significant difference between the SAb−/GAg − and SAb+/GAg+ groups (log-rank *p* = 0.003). The multivariable Cox proportional hazard analysis showed that age (HR = 0.968; 95%CI = 0.946–0.990; *p* = 0.005), SAb+/GAg+ versus SAb−/GAg− (HR = 0.387; 95%CI = 0.190–0.788; *p* = 0.009), SAb−/GAg+ versus SAb−/GAg− (HR = 0.398; 95%CI = 0.169, 0.939; *p* = 0.035), total renal chronicity score ≥2 (HR = 0.461, 95%CI: 0.277–0.766, *p* = 0.003), and IgA deposition (HR = 2.596; 95%CI = 1.227–5.492; *p* = 0.013) were all independently related (*p* < 0.05) to CR.

**Conclusions:**

The SAb and GAg status was an indicator of PMN prognosis. The patients with SAb−/GAg − had an increased likelihood of achieving CR than those with SAb−/GAg+ and SAb+/GAg+.

## Introduction

Membranous nephropathy is a common type of adult nephrotic syndrome with an incidence of around 10 per million population per year [1]. In China, membranous nephropathy prevalence has recently increased and became the leading pathological type of primary glomerular disease [2]. The majority of cases, approximately three quarters, occur spontaneously as idiopathic or primary membranous nephropathy (PMN). The secondary causes are associated with autoimmune diseases such as systemic lupus erythematosus, infection such as hepatitis B or hepatitis C, drugs, and cancer [1].

Membranous nephropathy is an antibody-mediated autoimmune disease that involves developing autoantibodies against antigens present on podocytes [3]. Patients with membranous nephropathy often present with proteinuria, and nephrotic syndrome occurs in 80% [4]. While some cases of PMN will spontaneously resolve, end-stage renal disease is a serious outcome in 41% of cases [3].

In 70% of cases of PMN, the anti-phospholipase A2 receptor (anti-PLA2R) antibody is associated with the disease [5]. The anti-PLA2R antibody has a higher positive rate in renal tissue, where it accumulates with IgG within immune deposits [6]. Therefore, some patients negative for anti-PLA2R antibodies might be positive for PLA2R antigen in renal tissue [7–9]. Anti-PLA2R antibody titers are associated with spontaneous remission and are important for monitoring the diagnosis and therapy of PMN [10–14]. It has been suggested that in the patients with anti-PLA2R antibody-associated PMN, the antibody titer might be used to predict prognosis, supposing that there is a lag between the immunological and clinical response [15]. Despite the fact that around 30% of patients with PMN are anti-PLA2R-negative, even in their renal tissue, little information is available on these patients [16]. Most studies using PLA2R to predict prognosis focused on the anti-PLA2R status of the patients, and few considered the glomerular PLA2R antigen [17].

The aim of this study was to examine the prognosis of patients with PMN according to their serum anti-PLA2R antibody (SAb) and glomerular PLA2R antigen (GAg) status.

## Methods

### Subjects

In this retrospective cohort study, patients diagnosed with PMN at Zhongshan People's Hospital from 1 January 2015 to 31 March 2018 were included. The exclusion criteria were the following: (1) <15 years old; (2) history of immunosuppressive therapy; (3) underwent kidney transplantation; or (4) incomplete follow-up information. The study was approved by the ethics committee of Zhongshan People's Hospital (approval number: K2019053). Informed consent was waived by the committee because of the retrospective nature of the study.

### Grouping

Patients were grouped according to their serum anti-PLA2R antibody and glomerular PLA2R antigen. There were three groups: serum anti-PLA2R antibody negative and glomerular antigen-negative (SAb−/GAg– group), serum anti-PLA2R antibody negative, and glomerular antigen-positive (SAb−/GAg+ group), and serum anti-PLA2R antibody positive and glomerular antigen-positive (SAb+/GAg+ group).

### Renal biopsy

All patients underwent renal biopsy before treatment. The pathological diagnosis of PMN was made using paraffin-embedded sections of 3 μm and using PAS, PASM, and Masson staining. Frozen sections were used for direct immunofluorescence to detect IgG, IgA, IgM, C3, C1q, and fibrin using a semi-quantitative method according to the fluorescence intensity (± to ++++). Transmission electron microscopy was performed using ultra-thin sections embedded in resin and stained with lead citrate and uranium acetate, to observe the ultrastructure, including the measurement of basement membrane thickness.

The histopathological characteristics of membranous nephropathy are the following: (1) light microscopy shows thickening of the glomerular basement membrane; (2) immunofluorescence shows that IgG and C3 are deposited along the glomerular capillary wall; and (3) electron microscope shows electron-dense deposits under the subepithelial and/or in the glomerular basement membrane. The individual scores for glomerulosclerosis (from 0 to 3), interstitial fibrosis (from 0 to 3), tubular atrophy (from 0 to 3), and arteriosclerosis (from 0 to 1) were calculated and then added together to provide the total renal chronicity score (TRCS) [18] (Supplemental Table S1). The TRCS was then used to grade the overall severity of the chronic lesions into minimal (0–1 total score), mild (2–4 total score), moderate (5–7 total score), and severe (≥8 total score). The glomerular lesions were graded into four stages based on the Ehrenreich and Churg’s classification criteria [19].

### Treatment

Treatment options included: non-immunosuppressive therapy and immunosuppressive therapy, including glucocorticoid + cyclophosphamide (CTX), glucocorticoid + cyclosporine (CsA), glucocorticoid + tacrolimus (FK-506), glucocorticoid + mycophenolate mofetil (MMF), and glucocorticoid + tripterygium wilfordii (a traditional Chinese medicine).

### Measurement of SAb and GAg

The peripheral venous blood of the patients was collected 3–5 days before the renal biopsy. The serum was separated by centrifugation and stored at −80 °C. The titers of serum anti-PLA2R antibody were measured by indirect immunofluorescence using the anti-PLA2R antibody detection kit (EUROIMMUN, Germany). Nonspecific fluorescence reaction with serum dilution below 1:10 was defined as negative, and specific fluorescence reaction with serum dilution of 1:10 and above was defined as positive. The positive results were expressed by a semi-quantitative titer.

PLA2R antigen in renal tissue was detected by immunohistochemistry using a rabbit polyclonal PLA2R antibody (Abcam, Cambridge, UK) according to standard protocols. To detect GAg presence, the percutaneous renal biopsy specimens were embedded in paraffin, sectioned at 3 µm, dewaxed, hydrated, heat-treated for 10 min at 120 °C, blocked with 10% FBS for 10 min, and incubated overnight at 4 °C with rabbit polyclonal antibodies against human PLA2R (Abcam, Cambridge, UK), followed by FITC-conjugated swine anti-rabbit IgG antibodies (Dako, Glostrup, Denmark) and reaction with 3,3′-diaminobenzidine [20]. The sections were dehydrated, sealed, and observed under light microscopy. A dark brown particle-like deposition along the glomerular basement membrane was defined as positive (Supplemental Figure S1).

### Data collection and follow-up

The following data were collected from the medical charts: demographic data including sex and age; baseline laboratory data including albumin, serum creatinine, estimated glomerular rate filtration (eGFR), and 24-h urinary protein; and renal pathological data including light microscopy, electron microscopy, and immunofluorescence.

The patients were followed routinely at 3, 6, and 12 months after treatment. Complete remission was defined as 24-h urinary protein <0.3 g or urinary albumin/creatinine <0.3 g/g, and serum albumin >40 g/L. Partial remission was defined as a 24-h urinary protein of 0.3–3.5 g or urinary albumin/creatinine of 0.3–3.5 g/g, and serum albumin >30 g/L. Doubling of the baseline creatinine values were considered as an indicator of decrease of renal function, and eGFR <15 mL/min/1.73 m^2^ was defined as development into end-stage renal disease (ESRD).

### Statistical analysis

The continuous variables were tested for normality using the Kolmogorov–Smirnov test. Data in accordance with a normal distribution were presented as means ± standard deviations (SD) and compared using analysis of variance (ANOVA) and Bonferroni *post hoc* test. Data with a non-normal distribution were expressed as medians (ranges) and compared using the Kruskal–Wallis test. Categorical variables were expressed in terms of frequencies and percentages and compared using the Chi-square test or Fisher's exact test. Cox univariable and multivariable regression analyses were used to screen for risk factors affecting prognosis (complete remission [CR]), so as to explore whether serum anti-PLA2R antibody and PLA2R antigen in renal tissue were independent risk factors for prognosis. Variables with *p* < 0.10 in the univariable analyses were included in the multivariable analysis, except for albumin because albumin levels were included in the definition of CR. The Kaplan–Meier survival analysis and log-rank test were used to compare the differences in prognosis among the three groups. A difference with *p* < 0.05 was considered statistically significant. Statistical analysis was performed using SPSS 19.0 (IBM, Armonk, NY).

## Results

### Baseline characteristics of the three groups

From 1 January 2015 to 31 March 2018, there were 135 eligible patients. Among them, 11 had a history of receiving immunosuppressive therapy, and 10 were lost to follow-up (Supplemental Figure S2). A total of 114 patients were included in the study: 10 patients (9%) in the SAb−/GAg– group, 23 patients (20%) in the SAb−/GAg+ group, and 81 patients (71%) in the SAb+/GAg+ group. No patient had SAb+/GAg–. There were no significant differences between the groups in terms of age or sex. Compared with the SAb−/GAg– group, the SAb+/GAg+ group had lower albumin levels (*p* = 0.023), higher 24-h urinary protein (*p* = 0.014), higher glomerular sclerosis ratio (*p* = 0.042), higher arteriosclerosis (*p* = 0.007), higher IgG deposition (*p* = 0.031), lower C1q deposition (*p* = 0.005), less advanced Ehrenreich and Churg’s stages (*p* = 0.016), and higher frequency of immunosuppressive treatment (*p* = 0.017), as shown in Table 1.

**Table 1.** Baseline data of patients with primary membranous nephropathy

	SAb-/GAg- (*n* = 10)	SAb-/GAg+ (*n* = 23)	SAb+/GAg+ (*n* = 81)	*p*
Demographic characteristics				
Age, years	45.70 ± 10.97	50.17 ± 15.16	46.98 ± 10.34	0.439
Male, *n* (%)	6 (60.0)	11 (47.8)	48 (59.3)	0.608
Laboratory characteristics				
Hematological parameters				
Albumin, g/L	29.07 ± 4.67	28.61 ± 6.29	25.88 ± 4.60	0.023^b^^,^^c^
Serum creatinine, μmol/L	73.50 ± 16.97	70.73 ± 24.27	78.53 ± 30.52	0.482
eGFR, ml/min/1.73 m^2^	100.26 ± 13.73	97.27 ± 24.13	97.39 ± 23.56	0.930
24 h urinary protein, g	1.51 (1.14,3.08)	2.01 (1.35,3.44)	3.66 (2.60,6.36)	0.007^b^^,^^c^
Pathological characteristics				
Glomerular sclerosis ratio, %	1.5 (0,5.8)	6.0 (0,13.0)	3.0 (0,8.5)	0.415
Renal tubular atrophy, *n* (%)	2 (20.0)	10 (43.5)	41 (50.6)	0.177
Interstitial fibrosis, *n* (%)	1 (10.0)	10 (45.3)	37 (45.7)	0.097
Arteriosclerosis, *n* (%)	7 (70.0)	9 (39.1)	19 (23.5)	0.007^b^
Mean TRCS	0.80	1.22	1.12	0.622
Positive immunofluorescence, *n* (%)				
IgG	8 (80.0)	23 (100)	80 (98.8)	0.031^a^^,^^b^
IgA	2 (20.0)	3 (13.0)	4 (4.9)	0.193
IgM	1 (10.0)	1 (4.3)	4 (4.9)	0.811
C3	1 (10.0)	3 (13.0)	15 (18.5)	0.674
C1q	4 (40.0)	1 (4.3)	3 (3.7)	0.005^a^^,^^b^
Fibrin	0	2 (8.7)	3 (3.7)	0.408
Thickness of basement membrane, nm	1480 ± 308	1391 ± 439	1325 ± 297	0.323
Churg’s stages, *n* (%)				0.016^b^
MN-I	0	4 (17.4)	8 (9.9)	
MN-II	5 (50.0)	13 (56.5)	64 (79.9)	
MN-III	5 (50.0)	6 (26.1)	9 (11.1)	
Immunosuppressive therapy				0.017^b^
No, *n* (%)	4 (40.0)	8 (34.8)	10 (12.3)	
Yes, *n* (%)	6 (60.0)	15 (65.2)	71 (87.7)	
Cyclophosphamide	3 (30.0)	4 (17.4)	48 (59.3)	
Cyclosporine	3 (30.0)	6 (26.1)	12 (14.8)	
Tacrolimus	0	3 (13.0)	11 (13.6)	
Mycophenolate mofetil	0	1 (4.3)	0	
Tripterygium wilfordii	0	1 (4.3)	0	

^a^ SAb-/GAg- vs. SAb-/GAg+, *p* < 0.05

^b^ SAb-/GAg- vs. SAb+/GAg+, *p* < 0.05

^c^ SAb-/GAg + vs. SAb+/GAg+, *p* < 0.05

Abbreviations: SAb = serum anti-phospholipase A2 receptor antibody, GAg = glomerular phospholipase A2 receptor antigen, eGFR = estimated glomerular filtration rate, TRCS = total renal chronicity score, MN = membranous nephropathy.

### Remission in the three groups during follow-up

The CR and partial remission (PR) rates at 12 months were significantly different among the three groups (*p* = 0.031), while the proportion of patients who achieved CR and PR at 3 and 6 months and experienced doubling of creatinine or ESRD were similar in the three groups (all *p >* 0.05, Table 2). Regarding spontaneous remission, the four patients without immunosuppressive treatment in the SAb–/GAg– group all achieved CR at 12 months. Four (50.0%) of the eight patients without immunosuppressive treatment in the SAb–/GAg+ group achieved CR, and four (50.0%) achieved PR at 12 months. The number of patients without immunosuppressive treatment in the SAb+/GAg+ group who achieved CR, PR, and no remission at 12 months was 6 (60.0%), 2 (20.0%), and 2 (20.0%), respectively.

**Table 2.** Clinical outcomes in patients with primary membranous nephropathy according to presence of serum anti-PLA2R antibody and glomerular PLA2R antigen.

	SAb−/GAg−	SAb−/GAg+	SAb+/GAg+	*p*
	(*n* = 10)	(*n* = 23)	(*n* = 81)	
Median follow-up time, months	14.10 (11.33,22.08)	10.90 (6.20,17.90)	16.06 (9.22,14.95)	0.577
3-month follow-up				0.279
CR rate	30.0%	8.7%	8.6%	
PR rate	30.0%	60.9%	53.1%	
6-month follow-up				0.176
CR rate	70.0%	39.1%	33.3%	
PR rate	30.0%	47.8%	53.1%	
12-month follow-up				0.031
CR rate	90.0%	52.2%	58.0%	
PR rate	10.0%	47.8%	32.1%	
Doubling of baseline creatinine levels or development of ESRD, *n* (%)^a^	0	1 (4.3)	3 (3.7)	0.681

PLA2R: phospholipase A2 receptor; SAb: serum anti-phospholipase A2 receptor antibody; GAg: glomerular phospholipase A2 receptor antigen; CR: complete remission; PR: partial remission; ESRD: end-stage renal disease.

^a^This is a composite endpoint that includes the development of any one of the two conditions or both.

**Figure 1.**
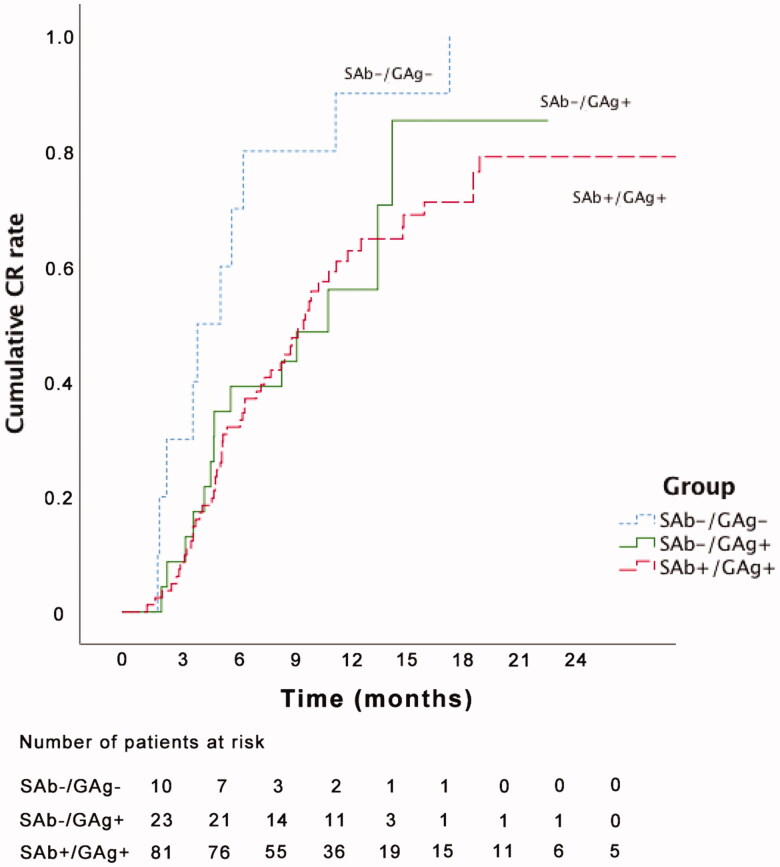
Survival curve and log rank test analysis of the three groups. SAb−/GAg − versus SAb+/GAg+, log-rank *p* = 0.003; SAb−/GAg − versus SAb−/GAg+, log-rank *p* = 0.061; SAb−/GAg + versus SAb+/GAg+, log-rank *p* = 0.835. CR: complete remission; SAb: serum anti-phospholipase A2 receptor antibody; GAg: glomerular phospholipase A2 receptor antigen.

When the Kaplan–Meier curves were plotted for cumulative CR, there was a significant difference between the SAb−/GAg − and SAb+/GAg+ groups (log-rank *p* = 0.003, Figure 1).

### Relationship between PLA2R and CR rate

The univariable analyses showed that age (hazard ratio [HR] = 0.978; 95% confidence interval [CI] = 0.958–0.999; *p* = 0.037), SAb+/GAg+ versus SAb−/GAg− (HR = 0.378; 95%CI = 0.191–0.746; *p* = 0.005), SAb−/GAg+ versus SAb−/GAg− (HR = 0.405; 95%CI = 0.179, 0.916; *p* = 0.030), albumin (HR = 1.051; 95%CI = 1.006–1.097; *p* = 0.025), total renal chronicity score ≥2 (HR = 0.661, 95%CI: 0.415–1.051, *p* = 0.080), and IgA deposition (HR = 2.359; 95%CI = 1.166–4.771; *p* = 0.017) were all significantly related (*p* < 0.10) to CR (Table 3).

**Table 3.** Univariable and multivariable Cox analyses of complete remission for patients with PMN, based on the PLA2R status (*n* = 114).

	Univariable			Multivariable		
	HR	95% CI	*p*	HR	95% CI	*p*
Age, years	0.978	0.958–0.999	0.037	0.968	0.946–0.990	0.005
Gender (male versus female)	1.197	0.762–1.881	0.434			
PLA2R status (versus SAb−/GAg−)						
SAb−/GAg+	0.405	0.179–0.916	0.030	0.398	0.169–0.939	0.035
SAb+/GAg+	0.378	0.191–0.746	0.005	0.387	0.190–0.788	0.009
Immunosuppressive therapy (yes versus no)	1.120	0.637–1.969	0.694			
Laboratory characteristics						
Hematological parameters						
Albumin, g/L	1.051	1.006–1.097	0.025			
Serum creatinine, μmol/L	1.005	0.997–1.012	0.217			
eGFR, ml/min/1.73 m^2^	1.001	0.990–1.012	0.839			
24 h urinary protein quantity, g	0.996	0.924–1.074	0.927			
Pathological characteristics						
Glomerular sclerosis ratio	0.997	0.969–1.026	0.824			
Renal tubular atrophy	1.189	0.762–1.856	0.445			
Renal tubular atrophy area ratio	1.018	0.974–1.065	0.430			
Arteriosclerosis	1.482	0.923–2.379	0.104			
Total renal chronicity score (≥2 versus 0–1)	0.661	0.415–1.051	0.080	0.461	0.277–0.766	0.003
Thickness of basement membrane, μm	1.302	0.772–2.197	0.322			
Immunofluorescence (versus (−))						
IgG (+)	0.409	0.099–1.685	0.216			
IgA (+)	2.359	1.166–4.771	0.017	2.596	1.227–5.492	0.013
IgM (+)	1.072	0.391–2.938	0.892			
C3 (+)	1.297	0.700–2.404	0.409			
C1q (+)	1.022	0.440–2.355	0.959			
Fg (+)	0.803	0.252–2.557	0.710			

Variables with *p* < 0.10 in the univariable analyses were included in the multivariable analysis. Cox proportional hazard models were used with SAb−/GAg − as the reference to find associations with CR.

PLA2R: phospholipase A2 receptor; CR: complete remission; PMN: primary membranous nephropathy; SAb: serum anti-phospholipase A2 receptor antibody; GAg: glomerular phospholipase A2 receptor antigen; HR: hazard ratio; eGFR: estimated glomerular filtration rate.

Cox proportional hazard models were used, with the SAb−/GAg − group as the reference, to find the effect of PLA2R status on the CR rate. The results are shown in Table 3. All variables with *p* < 0.10 in the univariable analyses were included in the multivariable model, except albumin levels, since this variable was included in the determination of the CR status. The multivariable analysis showed that age (HR = 0.968; 95%CI = 0.946–0.990; *p* = 0.005), SAb+/GAg+ versus SAb−/GAg− (HR = 0.387; 95%CI = 0.190–0.788; *p* = 0.009), SAb−/GAg+ versus SAb−/GAg− (HR = 0.398; 95%CI = 0.169, 0.939; *p* = 0.035), total renal chronicity score ≥2 (HR = 0.461, 95%CI: 0.277–0.766, *p* = 0.003), and IgA deposition (HR = 2.596; 95%CI = 1.227–5.492; *p* = 0.013) were all independently related (*p* < 0.05) to CR (Table 3).

## Discussion

This study aimed to compare the prognosis of patients with PMN according to their PLA2R status in terms of CR. The results showed that various factors were related to CR. In a Cox proportional hazard model adjusted for the variables that showed significant (*p* < 0.10) differences among the groups, the CR rate of the SAb−/GAg − group was significantly higher than that of the two other groups. Kaplan–Meier curves for cumulative CR demonstrated a significant difference between the SAb−/GAg − and SAb+/GAg+ groups. Therefore, these results indicate that along with various clinical factors related to CR, patients who had both negative serum antibody and tissue antigen for anti-PLA2R were more likely to achieve CR.

The patients in the SAb−/GAg − group showed less deteriorated disease markers than in the SAb+/GAg+ group, including albumin levels, 24-h urinary protein, glomerular sclerosis ratio, arteriosclerosis, IgG deposition, C1q deposition, Ehrenreich and Churg’s stage, and a requirement for treatment. The SAb−/GAg − group achieved the best prognosis of all three groups, with 12-month a CR rate of 90% and the fastest time-to response; the SAb+/GAg+ group had a 12-month CR rate of 58.0%; the SAb−/GAg+ group had a 12-month CR rate of 52.0%; those results could be expected based on the clinical and biochemical markers [4]. Nevertheless, the groping based on SAb and GAg had an effect on prognosis that was independent of the clinical and biochemical markers. The SAb−/GAg − group (i.e., non-PLA2R PMN) had the highest rate of remission. This is somewhat surprising because patients who are SAb−/GAg+ are typically thought of as having humoral remission and thus being on the way to clinical remission. Nevertheless, at baseline, the SAb−/GAg − group had much less severe disease than the two other groups. This has previously been reported in some, but not all studies of PMN [21,22]. Age ≥60 years, low serum albumin concentrations, and severe tubulointerstitial injury were identified as independent risk factors for the occurrence of ESRD in PMN patients [23]. Another study found that there were no significant differences in serum creatinine, albumin, or urine protein excretion between patients with PLA2R-associated and non-PLA2R-associated PMN [24]. The patients with non-PLA2R-associated PMN showed more abnormal serological tests and responded more quickly to immunosuppressive therapy [24]. Those studies support the results reported here.

The proportion of patients with serum positive for anti-PLA2R antibodies was 71%, similar to previous studies that reported that around 70% of patients were serum positive for anti-PLA2R antibodies in the USA [10] and Asia [12], but different frequencies have been seen in Japanese patients, with 53% of patients with PMN having positive serum results [25], and 52% in a German study [26]. One Chinese study found that 58.8% of patients had serum positive for anti-PLA2R antibodies [27], while another showed that the percentage of patients with PMN who were positive for anti-PLA2R antibodies was 56.3% [7]. Therefore, it appears that there are some differences among different populations. Larger studies with standardized testing procedures are needed to evaluate these differences fully. Our study also found that 20% of the patients were serum negative for anti-PLA2R antibodies but were positive for PLA2R antigen in renal tissues (SAb−/GAg+ group). Overall, 91% of the patients with PMN had detectable PLA2R in the glomeruli. This rate was similar to the levels seen in the study by Pang et al. (98%) [27] but was higher than that observed by Hihara et al. (58%) [8].

Previous studies mainly focused on the antibody status of patients with PMN and showed that an increase in anti-PLA2R antibodies in serum predicted kidney dysfunction [7] and that a decrease in anti-PLA2R antibodies preceded remission [9]. Anti-PLA2R antibodies were identified as an independent risk factor for developing chronic kidney disease stage ≥3 and for not reaching spontaneous remission [28]. Some studies had also included glomerular PLA2R antigen in their analysis. In addition to serum anti-PLA2R antibody levels persisting in patients who did not achieve remission and is significantly decreased in patients who achieved remission, one study showed that sustained glomerular antigen deposits also correlated with disease relapse [29]. On the other hand, a study that investigated prognosis in terms of proteinuria found that anti-PLA2R antibody was correlated with this outcome, but glomerular PLA2R antigen was not [17]. They came to a different conclusion to ours and suggested that the anti-PLA2R antibody but not glomerular PLA2R antigen predicts outcome [17]. Our study suggested that both anti-PLA2R antibody and glomerular PLA2R antigen were important for prognosis prediction.

As renal biopsy is an invasive procedure that may induce complications, it has been suggested that serum anti-PLA2R antibody status alone might be able to predict prognosis [15]. Nevertheless, the results of our study supported the view that this can only be useful in patients with PMN with preserved kidney function who are anti-PLA2R antibody-positive [15,30]. For patients with PMN who are negative for anti-PLA2R antibodies, there might be two conditions: one is PLA2R-related (SAb−/GAg+), and the other one is non-PLA2R-related (SAb−/GAg−). Because our data suggested that the prognosis of the patients in these two groups was significantly different, patients who are negative for PLA2R antibodies should undergo a renal biopsy to predict prognosis better. The Cox proportional hazard modeling showed that when adjusted for baseline demographics, treatment, laboratory, and pathological data, the CR of the SAb−/GAg − was superior to that of the SAb−/GAg+ and SAb+/GAg+ groups. Thus, it may be possible to increase the prediction of CR rate by including pathological data to the anti-PLA2R antibody status.

It is possible that the patients in the SAb−/GAg − group in this study had a different mechanism of developing membranous nephropathy than in those with anti-PLA2R antibodies. Autoantibodies to other antigens may be important in this subset of patients with PMN [31]. One study showed that 15 of 154 patients with PMN had circulating autoantibodies to thrombospondin type-1 domain-containing 7 A (THSD7A) [20]. Another study suggested a prevalence of 2.6% [32]. The mechanism appears to involve anti-THSD7A autoantibodies binding to podocyte-expressed THSD7A, inducing proteinuria, and the morphologic hallmarks of PMN [33]. A preliminary study suggested that the THSD7A antibody titer might also be associated with disease activity [15]. Even though the patients with proven secondary membranous nephropathy (SMN) were excluded from the study, there is a possibility that some patients with SMN were included because no secondary cause was identified. The diagnosis of PMN was based on the patient's medical history and laboratory tests to exclude SMN, and on a renal pathology that did not explicitly suggest SMN. Nevertheless, secondary causes of membranous nephropathy may manifest later as the disease develops or may have been detected by more advanced detection methods that were not used by the attending physicians. Therefore, for patients with membranous nephropathy without a clear pathogenic antigen, it could be necessary to search for possible secondary causes actively.

There were some significant differences in treatment strategy identified among the three groups of patients. It has been suggested that monitoring serum anti-PLA2R antibody titer may assist in determining when to initiate the administration of immunosuppressive agents and in evaluating treatment efficacy [17]. This was reflected in our study by fewer patients in the SAb−/GAg − group receiving immunosuppressive therapy (60.0%) than in the SAb−/GAg+ group (65.2%) and the SAb+/GAg+ group (87.7%). Although our result suggested that immunosuppressive therapy was not associated with CR, it was suggested that immunosuppression must be undertaken and monitored carefully because while it can prevent renal progression and all-cause mortality in PMN patients, there are concerns over the substantial toxicity of some immunosuppressive treatments [34].

This study has some limitations. First of all, the number of cases in the SAb−/GAg − group was quite small. A larger study would provide more evidence for our results. This was a retrospective study, and patients were followed in an inconsistent frequency, so there may be some biases in the results. As this was not a clinical trial with pre-defined follow-up, but a retrospective study based on the actual patients' management, the patients were followed based on each physician's judgment based on each patient's condition. The choice of immunosuppressive agents was mainly made by the attending physician. Since this is a retrospective study based on patient charts, the rationale for selecting one treatment strategy over another was not indicated in the charts and could not be analyzed. The KDIGO guidelines are routinely followed, but the traditional Chinese medicine is deeply rooted in the Chinese culture and is often more affordable than conventional treatments, but only one patient received Tripterygium wilfordii 20 mg tid. The changes in serum anti-PLA2R antibody titer were not monitored, and this might have an influence on prognosis. In addition, data on relapse was not collected. Finally, patients with SAb − and who did not undergo renal biopsy could not be included in the present study, and some cases were probably missed.

In conclusion, this study compared patients with PMN according to their serum anti-PLA2R antibody and renal tissue PLA2R antigen status. The results showed that along with various clinical factors related to CR, patients who were both serum antibody and glomerular antigen-negative for anti-PLA2R were more likely to achieve CR. Further large-scale multicenter prospective studies are needed to confirm this conclusion.

## Supplementary Material

Supplemental Material

Supplemental Material

Supplemental Material
