# Supplementary figures and images for: Outcomes of primary membranous nephropathy based on serum anti-phospholipase A2 receptor antibodies and glomerular phospholipase A2 receptor antigen status: a retrospective cohort study
Source: Ren Fail. 2020 Jul 17;42(1):675–83. doi: 10.1080/0886022X.2020.1792315 (PMC7470143; doi:10.1080/0886022X.2020.1792315)

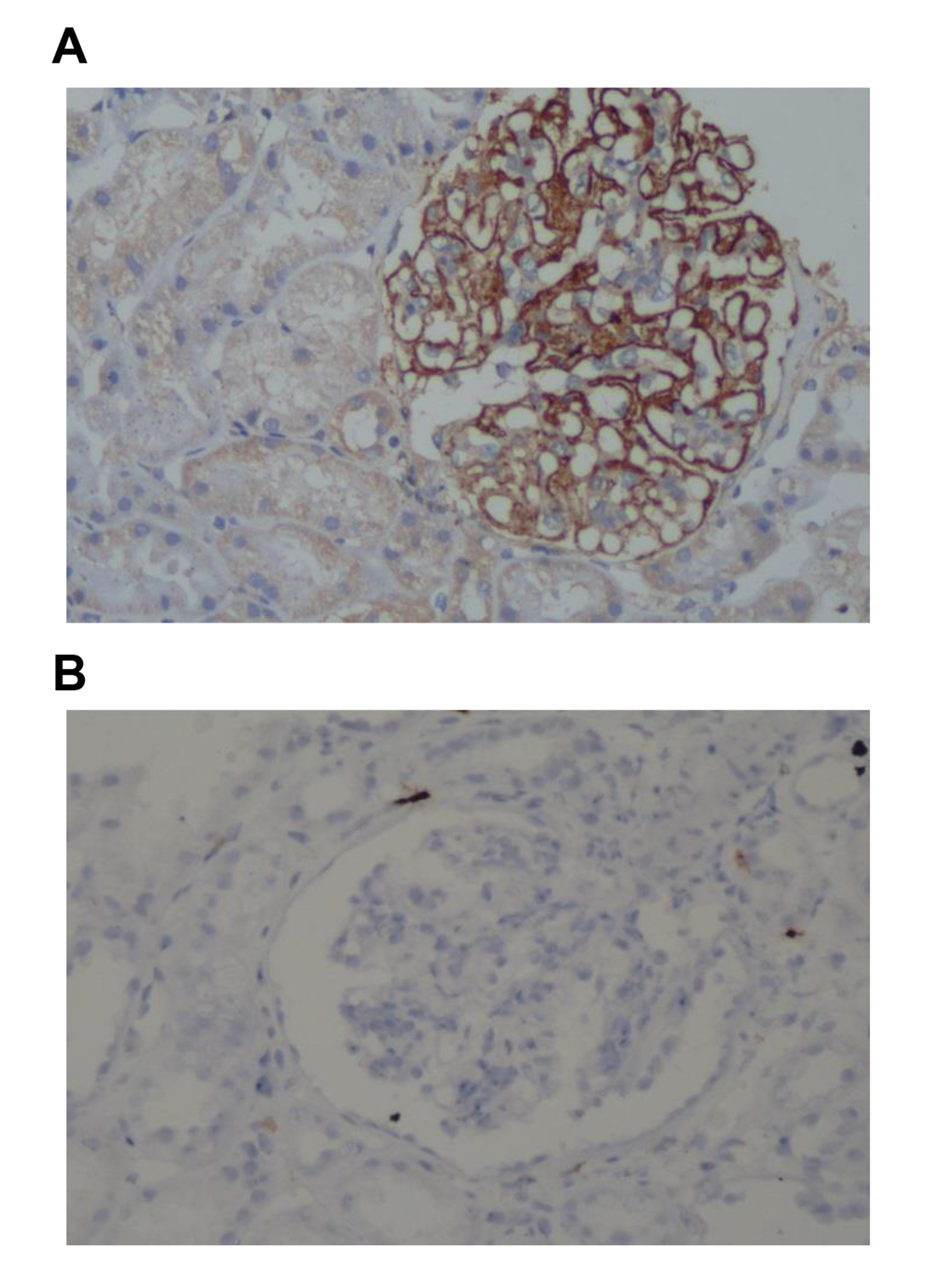

Supplement: Supplemental Material [file IRNF_A_1792315_SM2225.tif]

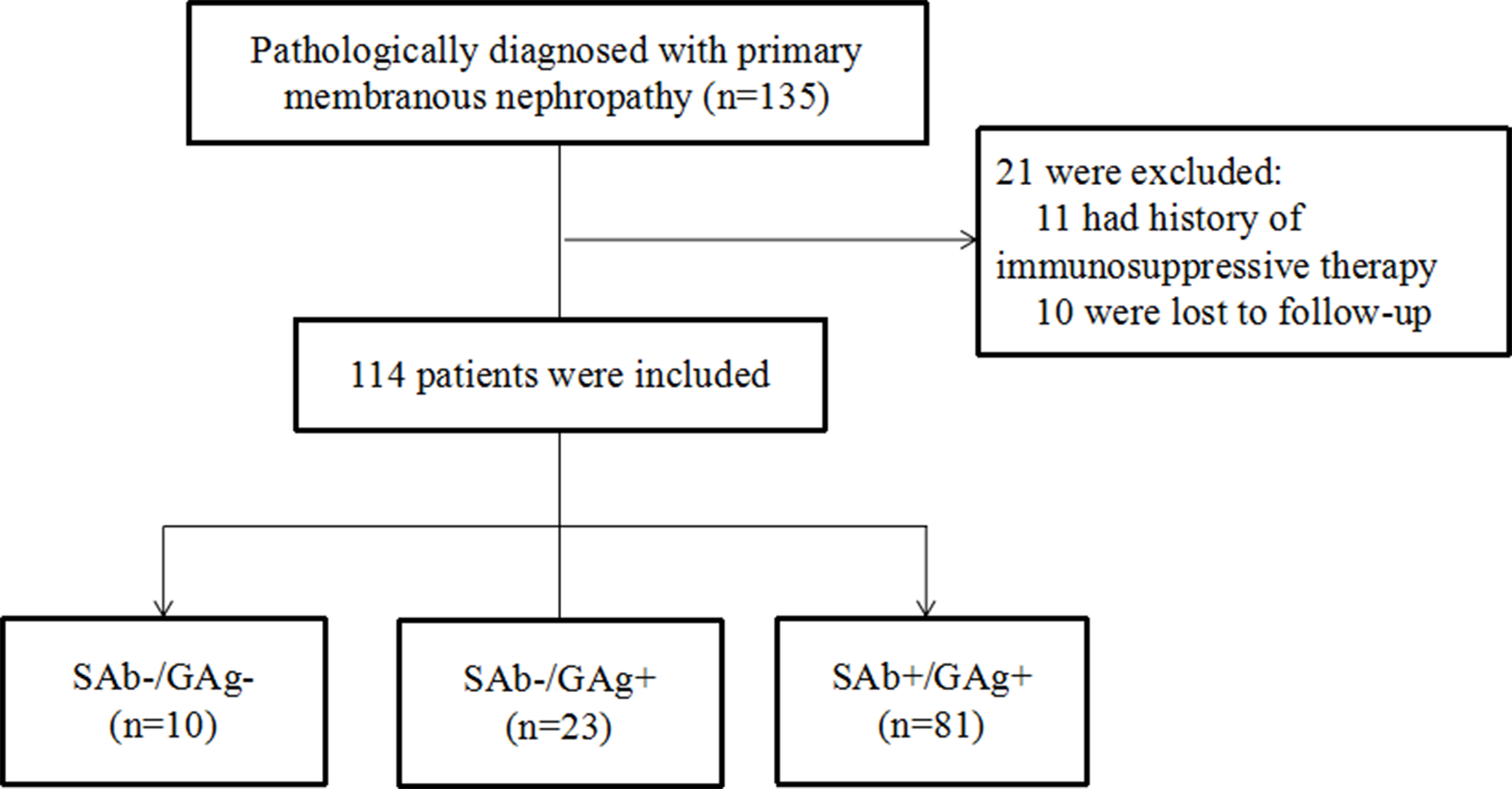

Supplement: Supplemental Material [file IRNF_A_1792315_SM2224.tif]
