# Supplementary material for: Outcomes of primary membranous nephropathy based on serum anti-phospholipase A2 receptor antibodies and glomerular phospholipase A2 receptor antigen status: a retrospective cohort study
Source: Ren Fail. 2020 Jul 17;42(1):675–83. doi: 10.1080/0886022X.2020.1792315 (PMC7470143; doi:10.1080/0886022X.2020.1792315)
Supplement: Supplemental Material [file IRNF_A_1792315_SM2187.pdf]

Supplemental Table S1. Scoring of the chronic lesions in individual renal tissue compartments

Tissue compartment [a]	Score			
	0	1	2	3
Glomerulosclerosis (GS score)	<10%	10-25%	26-50%	>50%
Interstitial fibrosis (IF score)	<10%	10-25%	26-50%	>50%
Tubular atrophy (TA score)	<10%	10-25%	26-50%	>50%
Arteriosclerosis (CV score)	Intimal thickening < thickness of media	Intimal thickening ≥ thickness of media		

[a] The GS score includes the percentage of glomeruli with global and segmental sclerosis and ischemic glomeruli; IF and TA score includes the percentage of renal cortex involved by interstitial fibrosis and tubular atrophy, respectively; CV score includes the severity of arteriosclerosis determined by the extent of thickening of the intima.
